# Use of Artificial Neural Networks and NIR Spectroscopy for Non-Destructive Grape Texture Prediction

**DOI:** 10.3390/foods11030281

**Published:** 2022-01-20

**Authors:** Teodora Basile, Antonio Domenico Marsico, Rocco Perniola

**Affiliations:** Centro di Ricerca Viticoltura ed Enologia (CREA-VE), Consiglio per la Ricerca in Agricoltura e l’Analisi dell’Economia Agraria, Via Casamassima 148, 70010 Turi, BA, Italy; adomenico.marsico@crea.gov.it (A.D.M.); rocco.perniola@crea.gov.it (R.P.)

**Keywords:** PCA, ANN, PLS, MC-UVE, β coefficients, R statistics, table grape

## Abstract

In this article, a combination of non-destructive NIR spectroscopy and machine learning techniques was applied to predict the texture parameters and the total soluble solids content (TSS) in intact berries. The multivariate models obtained by building artificial neural networks (ANNs) and applying partial least squares (PLS) regressions showed a better prediction ability after the elimination of uninformative spectral ranges. A very good prediction was obtained for TSS and springiness (R^2^ 0.82 and 0.72). Qualitative models were obtained for hardness and chewiness (R^2^ 0.50 and 0.53). No satisfactory calibration model could be established between the NIR spectra and cohesiveness. Textural parameters of grape are strictly related to the berry size. Before any grape textural measurement, a time-consuming berry-sorting step is compulsory. This is the first time a complete textural analysis of intact grape berries has been performed by NIR spectroscopy without any a priori knowledge of the berry density class.

## 1. Introduction

Sensory texture characteristics play a key role in the customer’s perceived quality of fresh table grape [1,2]. Texture characterization is conventionally performed either by sensory or instrumental texture analysis. Grape berries are usually sorted based on their density before the instrumental texture analysis. Berries belonging to the same class (a small range of density values) are considered technical replicates and are used to compute statistical parameters such as mean and standard deviation [3]. In the sensory texture analysis, a trained sensory panel evaluates the product following an experimental design. The application of specific statistical analyses allows the interpretation of the outcome [4]. Both sensory and instrumental texture analysis are destructive methodologies. This means they do not allow for any technical repetition of the measurement nor further analysis of the same sample. In this scenario, the use of a fast and non-destructive analytical technique that allows a multi-component analysis looks like a promising alternative.

Near-infrared (NIR) spectroscopy associated with multivariate data analysis has been widely employed for fruit and vegetable analysis [5,6]. Chemometric techniques are applied to extract the data from the recorded spectra, which are employed to build a prediction model for various parameters (e.g., sweetness, acidity, antioxidant content, and so on), in conjunction with data from reference methods. 

The NIR technique is a secondary method that strongly relies on the accuracy and precision of the data obtained with the reference methods when it is used for prediction purposes [7,8]. The application of NIR spectroscopy to the prediction of textural properties could also be hindered by the poor accuracy and precision of conventional analytical methods used for texture properties. Indeed, the primary method employed in this work was the instrumental Texture Profile Analysis (TPA), which requires an *a* priori densimetric sorting of the berries since it does not allow for technical replicates and relies on a standard deviation value obtained from a pool of samples within the same density range. 

NIR spectroscopy is a sensitive analytical technique, and it can even identify the geographical origin of samples since it is able to differentiate among samples based on the effects of different cultural systems and different pesticide treatments, and so on [9].

It is evident how a selection of the samples before the analysis would greatly simplify the procedure and increase the prediction performances of the models. However, not only are these preliminary steps time-consuming but, moreover, a model built only for berries belonging to a selected range of density has limited practical application, since the actual need is to characterize berries randomly picked in the vineyard.

This work aimed to build optimal prediction models for texture parameters of grapes of the same variety, grown in the same vineyard but collected from three different distant blocks without any a priori knowledge on the ripening stage (i.e., density class). 

NIR spectroscopy is usually coupled with Partial Least Squares (PLS) regression analysis that allows the development of linear regression models for the prediction of the parameters of interest. However, linear regressions models are not always able to effectively predict parameters that are not linked to a specific compound or class of similar compounds (e.g., different sugar molecules for sweetness), but rather to a complex combination of factors (e.g., water content, types of pectins, and so on), which is not yet clearly known, as it is for texture-related parameters [10,11]. In these cases, the use of non-linear models in the creation of an optimal prediction model, such as artificial neural networks (ANNs), has been shown as advantageous over PLS models. The theory and the application of ANNs in modeling chemical data have been exhaustively reported in the literature [12]. ANNs are powerful methods with pattern recognition abilities. These abilities make them perfect for the extraction of quantitative information from large spectroscopic databases where non-linearity is inherent due to complex biological, environmental, and instrumental variations. The efficiency of ANN methods is undisputed, however, the network implementation, method setup, training, and estimation of parameters are relatively complex compared to linear regression methods [13].

The sugar content of fruits is a parameter usually well-predicted by NIR since sugar molecules possess NIR active groups and are among the most abundant compounds in fruits. The total soluble solids (TSS) index is a measure of the density (mass/volume) of all soluble solids. The TSS value mainly reflects the sugar content in grapes at harvest. TSS prediction was performed following the same procedure implemented for the texture parameters as a comparison. To our knowledge, this is the first time that texture parameters have been predicted from NIR spectra of intact berries with sufficient accuracy and precision.

## 2. Material and Methods

### 2.1. Grape Samples

Regal Seedless grape berries were harvested from the experimental vineyards of CREA Research Centre for Viticulture and Enology of Turi, Southern Italy (40°57′26″ N; 17°00′26″ E) in 2018. Fifteen bunches were collected at harvest from three blocks in different areas of the vineyard. For each block, 3 plastic bags containing 50 berries each were placed in a cold store at 0 °C, until the analysis. A total of 30 berries were randomly picked from each plastic bag and left at room temperature (20 °C) for 2 h. Each berry was weighed on a top-loading balance (accuracy of ±0.001 g). The berries were then sorted according to their density by flotation in different saline solutions following the procedure described elsewhere [14]. The densimetric sorting described here was performed only in order to obtain standard deviation values, and the NIR analysis was performed without any knowledge of the density class of the single berries. Each berry was shortly washed with distilled water and gently tapped with paper before the NIR measurement. After spectra acquisition, a TPA was performed followed by a TSS measurement.

The 270 berry samples were measured over several days. Two different operators performed the TPA and NIR analyses in parallel. The dataset used for the external validation is comprised of samples randomly taken from the whole dataset, and therefore is composed of berries measured in a span of time. This protocol ensures the robustness of the models.

### 2.2. TSS Measurement

A total soluble solids (TSS, °Brix) measurement, in triplicate at 20 °C using a digital refractometer Atago PR1 (Atago Co., Tokyo, Japan), was performed following the official OIV method [14].

### 2.3. Instrumental Texture Analysis

The TPA was performed on each berry using an XforceP texture analyzer (Zwick/Roell GmbH & Co., Ulm, Germany) equipped with the Zwick Roell software package (testXpert II Zwick/Roell, ver. 3.31, Ulm, Germany). On each berry, a double compression test was performed. The individual berries were placed in their equatorial position on a metal base (first probe) and underwent a double compression with a 35 mm P/35 flat cylindrical probe (second probe) under 20% deformation. The waiting time between the two compressions was 2 s and the test speed was 1 mm/s. From the force–time curve obtained, the software automatically calculated the following parameters: hardness (BH in N), springiness (BS in mm), cohesiveness (a-dimensional, BCo), gumminess (BG in N, as BH × BCo), and chewiness (BCh in mJ, as BH × BCo × BS) [15]. The equatorial diameter of each berry was also provided by the software as the distance between the two probes when the second probe touched the surface of the berry.

### 2.4. NIR Spectral Data

NIR spectra of berry samples were acquired in diffuse reflection mode with a TANGO FT-NIR (Fourier Transform Near-Infrared) spectrometer produced by Bruker, Germany. The spectral scanning range was 12,000–4000 cm^−1^ (833–2500 nm), with 8 cm^−1^ resolution and 64 scans. Each berry was scanned three times, moving the berry in different positions (three different berry faces), and the resulting mean spectrum was used to represent each sample. A background spectrum was automatically recorded, before each sample, while both temperature and humidity were kept constant.

### 2.5. Statistical Analysis

The statistical procedures described in detail in the following paragraphs, including pre-treatments of the original spectra, Mahalanobis distance calculation, principal component analysis (PCA), calibration, cross-validation, and external validation of prediction models obtained with PLS regression and ANN, were performed using the open-source R statistical software (R version 3.6.3 (29 February 2020) Copyright © 2020 The R foundation for Statistical Computing [16]). The R packages used are listed in alphabetical order, as follows: chillR [17], ClassDiscovery [18], enpls [19], ggbiplot [20], ggplot2 [21], keras [22], mdatools [23], Metrics [24], prospectr [25], signal [26], and SimDesign [27].

### 2.6. Pre-Treatment Selection, PCA, and Outlier Removal

The original spectra were pre-treated with the preprocessing steps conventionally applied to spectroscopic data: scatter correction (Standard Normal Variate (SNV), Multiplicative Scatter Correction (MSC)), noise reduction by smoothing (Savitzky–Golay smoothing with different window width, polynomial, and derivative order), and scaling (mean center) [28]. Derivatives can be very useful in NIR spectroscopy for removing some of the extraneous signals from the spectra. However, since derivatives tend to increase the noise, we applied the Savitzky–Golay algorithm for smoothing after derivatization. Concerning the derivative degree, we even performed third and fourth derivatives. However, as expected the higher-order derivatives did not provide any improvement. A PCA was performed on each pre-treated spectral dataset. A summary of the combination of pre-treatments applied together with the cumulative variance explained by the first two PCs is reported in Supplementary Figure S1. 

The pre-treatments to be applied before the PLS modeling were selected based on the highest amount of cumulative variance explained. However, pre-treatments leading to a PCA discrimination of the sample based on the experimental design were discarded. The application of mean centering or SNV as pre-treatments resulted in the samples forming a quite compact group in the center of the PCA plot, with only a few detected as outliers. The application of smoothing (despite the polynomial exponent or the sampling space) resulted in a spread of the samples in the PCA plot with a higher number of spectra recognized as outliers. In some cases, the samples were divided into three groups, resembling the three sampling sites used in the experimental box plot design. Since those pre-treatments are probably able to discriminate the sample mainly based on the experimental design in the field, the smoothing was discarded.

Based on the selection criteria described above, the selected pre-treatments were: mean center and SNV followed by mean center. The outlier detection on each of the selected pre-treated spectral datasets was performed by calculating the Mahalanobis distance for spectral data. A PLS analysis was performed on each of the selected spectral datasets to check for outliers and extreme objects based on the computation of the critical limits with the robust approach (which utilizes median and inter-quartile range instead of mean and standard deviation, as in the data-driven approach) [29]. The outliers that were eventually found were also removed from the dataset.

### 2.7. Development of the Prediction Models

#### 2.7.1. Βeta-Coefficients and MC-UVE

Two wavelength selection methods were applied to eliminate the relatively uninformative variables: the Monte Carlo uninformative variable elimination (MC-UVE), and the high β regression coefficients [30]. This step was performed to reduce the number of variables used to build the PLS model and to train the ANN. 

MC-UVE is a frequently applied variable selection method that combines the Monte Carlo strategy with the uninformative variable elimination method. Wavenumbers with large effects are important for predicting the parameter. However, predictors with large size but also large variance (their importance may vary on different subsets of the samples) were discarded [31]. The MC-UVE method builds a large number of models with randomly selected calibration samples at first, and then evaluates the “importance” of each variable with a value of stability of the corresponding coefficients in these models. Variables with poor stability are known as uninformative (their contribution to models is small) variables and are eliminated [32].

The β regression coefficients were obtained from PLS models as previously described [33]. Confidence intervals, statistical significance, and other statistics for the coefficients were calculated using the Jack-Knife method. The wavelengths that corresponded to the statistically significant highest absolute values of β-coefficients and the MC-UVE selected ones were used as data inputs to establish multiple linear regression models using R.

#### 2.7.2. Data Normalization and Split into Training and Test Sets

In the training of a neural network, a common practice is to normalize the input data (mean close to 0). Normalized data generally increase the learning rate and lead to faster convergence. A min-max normalization was applied to scale the input variables in the interval [0,1]. For each selected pre-treatment, the Kennard–Stone algorithm split the normalized data into a training set composed of 80% of the samples and a test set containing the other 20%. The training set was used to calculate and optimize the regression models with cross-validation. The test set was employed to evaluate the predictive ability of the model.

#### 2.7.3. PLS Models

A multivariate calibration was performed using the PLS regression (SIMPLS algorithm) with a leave-one-out cross-validation. The optimal number of components was calculated for the different number of components and through predictions. The detection and removal of the outliers performed were previously described [34]. Shortly, a robust approach, insensitive to small and larger deviations, which utilizes the median and inter-quartile range instead of mean and standard deviation, was used for computing the critical limits for residual distances [29]. After the outliers’ detection and removal step, a classic approach, namely the data-driven approach, based on classical estimators (statistical moments), was used to build the final model [35]. The performance of a PLS regression can be improved by selecting characteristic wavelengths (holding sample-specific or component-specific information) from the full spectrum. Eliminating uninformative variables can be useful to build better quantitative calibration and prediction models [31]. Several methods have been developed, and are described in the literature. The ones performed on our data are explained in detail in the previous paragraphs. 

#### 2.7.4. ANN Structure

The structure of the feed-forward fully connected neural network consisted of one layer for each of the three classes. We found that increasing the number of hidden layers resulted in a worsening of the prediction of our parameters. The number of neurons in each layer was: number of predictors + 1 for the input, half of the input data for the hidden one, and one neuron for the output layer since we were performing a regression analysis. In summary, the ANN configuration was input:hidden:output, *n* + 1:(*n* + 1)/2:1, where *n* is the numeric vector representing the selected wavenumbers for each NIR spectrum. The activation function for the first and second layer was a Rectified Linear Unit (ReLU) activation function with a He normal initialization, commonly used for weight initialization parameters with ReLu activation. An L1 regularization was applied to both the input and the hidden layers to reduce over-fitting by keeping network weights small. In the training procedure of the ANN model, we used the Adam optimizer, the mean squared error as a loss function (the function to minimize during optimization), and the mean absolute error to monitor the training. The training was structured into 1000 epochs, with a batch size of 32 and a validation split of 0.2 (80% of the data was used to train and 20% to test the model).

## 3. Results and Discussion

### 3.1. Raw NIR Spectral Analysis

In a previous article, samples from the same vineyard subjected to identical treatments were collected, sorted by density, and analyzed with PLS and iPLS regressions for hardness prediction [11]. In order to evaluate the ability of the NIR technique to overcome the differences induced by different treatments and avoid the sorting step, in this work, samples collected from vines grown with three different practices belonging to different areas of the same vineyard were analyzed.

**Figure 1:**
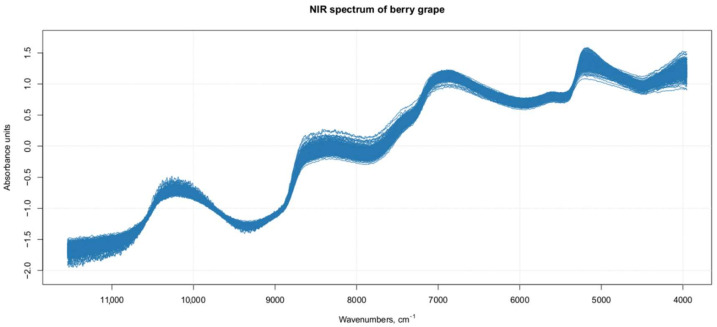
Spectra of single berries (each spectrum is a mean of three repetitions of different berry faces).

Figure 1 shows the NIR original and raw spectra of 270 Regal berries. The spectrum of each berry is a mean of three spectra recorded on different berry faces. Water signals are dominant in the NIR spectra of grape, since water is the main component of this fruit, and show very strong absorption bands in the NIR region. Another important component of grapes is sugar [36]; however, the molecular bonds of the different sugar molecules, which are active in the NIR region, are often placed in the same region as the major absorption peaks of water. The comparison of the main peaks observed in our spectra with literature data allowed a tentative attribution of the signals to molecular bonds of specific compounds. Wavelengths near 950 and 1460 nm (10,526 and 6849 cm^−1^) can be related to the third O–H overtone from water absorption. The absorptions at 1450 and 1950 nm (6896 and 5128 cm^−1^) were related to the first overtone of the O–H stretch and the combined stretch and deformation of O–H groups from water and glucose. Absorptions at 1690 nm (5917 cm^−1^) can be related to the first overtone of the C–H_3_ stretch, while those at 1750 nm (5714 cm^−1^) relate to the first overtones of the C–H_2_ and C–H stretches in glucose and water. Absorption bands near 1200 nm (8333 cm^−1^) are related to sugars. Variations near 990 nm (10,101 cm^−1^) are associated with the O–H stretch second overtones from organic acids and various sugars. The absorption at 2260 nm (4424 cm^−1^) is likely related to a combination of C–H and O–H stretch overtones, the latter from glucose, and absorption at 2302 nm (4344 cm^−1^) is primarily related to C–H combination vibrations (CH_3_ and CH_2_) from carbohydrates and organic acids [33,37,38,39].

### 3.2. Prediction of Unknown Samples

The model created with the known samples can then be used for the prediction of the same parameters of unknown samples. The analysis performed in this work faced some challenges linked to the nature of the samples, the precision and accuracy of the primary methods, and the sensitivity of the NIR technique. Intact berries are highly inhomogeneous samples. This natural characteristic of the berries produces random noise in NIR spectra that can be hard to detect and remove. 

### 3.3. TSS Model

**Figure 2:**
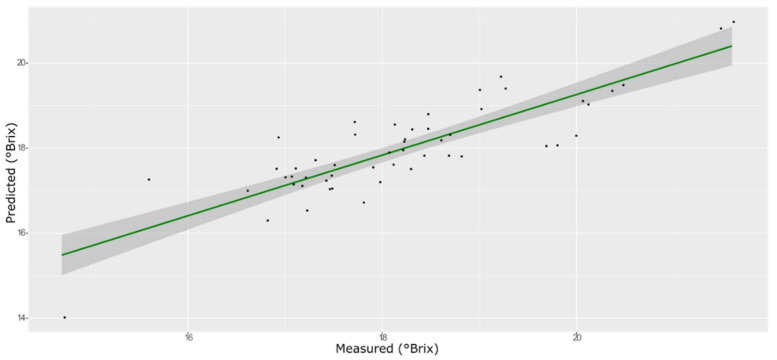
PLS model for TSS on the test set, using the full spectral range.

**Figure 3:**
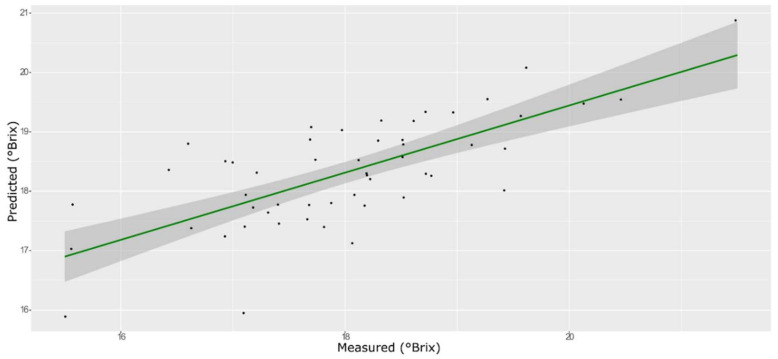
PLS model for TSS on the test set, using the selected wavenumbers.

**Figure 4:**
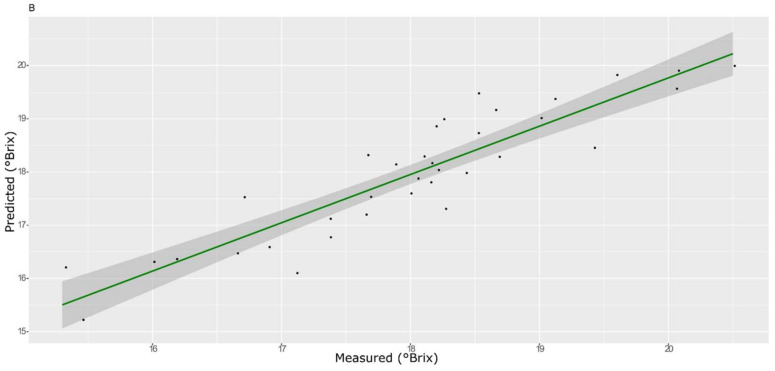
ANN model for TSS on the test set.

**Figure 5:**
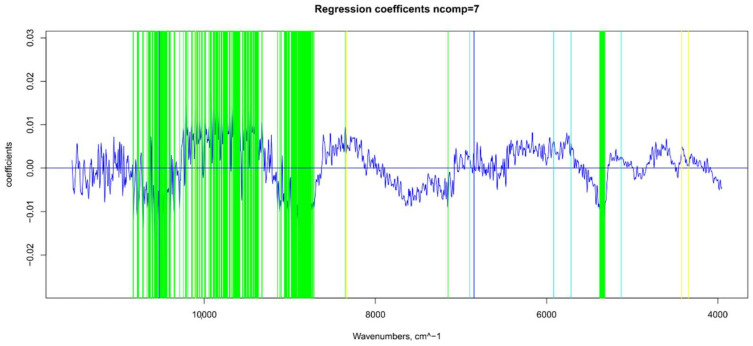
Βeta-coefficient values after outlier removal and mean center: values selected for ANN (in green), main NIR peaks for water (in blue), sugar and water (in light blue), and sugar and organic acids (in yellow).

For the sugar content prediction model, the selected pre-treatment was an SNV followed by a mean center. From high β-coefficients selection, 290 wavenumbers with a *p*-value < 0.05 were retained as input data. The prediction capability of the models on the training and test sets was evaluated by the root mean square error (RMSE), coefficient of determination (R^2^), bias, and residual predictive deviation (RPD, ratio of standard error of performance to standard deviation) index. The performance of the PLS models did not improve after the removal of the relatively uninformative variables (Figure 2 and Figure 3 and Supplementary Figures S1 and S2). The ANN model gave the best prediction for TSS with the better fit (R^2^ 0.82), higher RPD (over 2), smaller bias, and smaller RMSE (Figure 4 and Supplementary Figure S3, and Table 1). The β selection of optimal wavebands for the prediction of TSS in grape berries resulted in better performing models compared to the MC-UVE. A comparison between the wavenumbers selected with the two methods shows how the very different spectral areas were chosen. Probably, the selection was based on *β*-coefficient extracted spectral areas, which more effectively described our samples. The *β*-coefficient plot is shown in Figure 5. The selected wavenumbers do not include the spectral areas in which the overtones of sugars are usually found. The peak selection criteria commonly followed are: wavelengths should have a statistically significant large absolute regression coefficient value and be in specific peaks and valleys of the regression coefficient curve [40]. Selecting the wavelengths which contribute to the investigated attribute of a sample should increase the prediction of the attribute itself. An additional ANN model was created adding sugar-related statistically significant (*p* < 0.005) signals to the set of predictors, however, the prediction ability decreased (302 predictors, test set model: R^2^ 0.4513, RMSE 0.97, bias 0.396, and RPD 1.38). In NIR spectra, a specific attribution to a class of compounds is not possible since signals are produced by functional groups found in different molecules. We hypothesized that the contribution to those spectral areas in which sugar overtones are usually reported in the literature was mainly attributable to compounds other than sugars (i.e., water and organic acids) for our samples.

The good correlation between the optical data and this ripening parameter confirms what has been previously found for both table and wine grapes [41,42].

### 3.4. Springiness

The model obtained with an SNV pre-process afforded the best prediction for BS using both PLS and ANN. A model built without any information about berry weight or size led to models with low prediction ability (data not shown). Due to the known strong influence of berry size on the TPA parameters, the instrumental outcome of the texture profile analysis is often normalized with the berry diameter or the berry volume [43]. Therefore, we used the equatorial diameter, which can be easily measured with a caliper as an additional factor.

**Figure 6:**
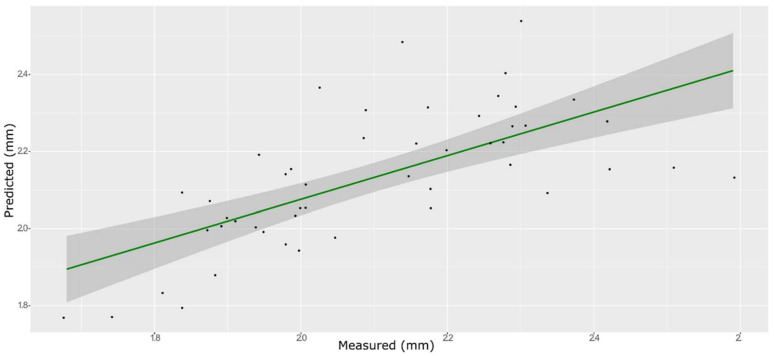
PLS model for BS on the test set, using the full spectral range.

**Figure 7:**
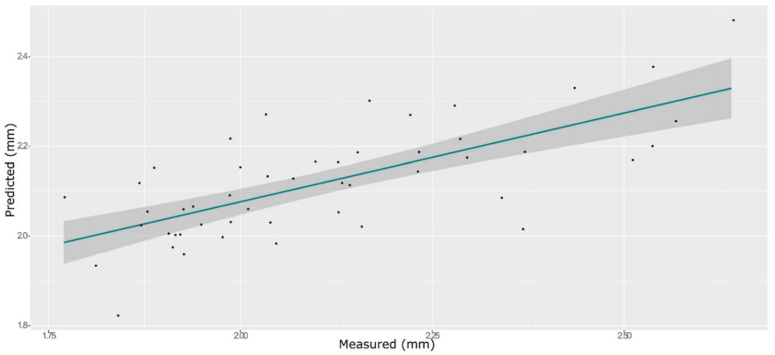
PLS model for BS on the test set, using the selected wavenumbers.

**Figure 8:**
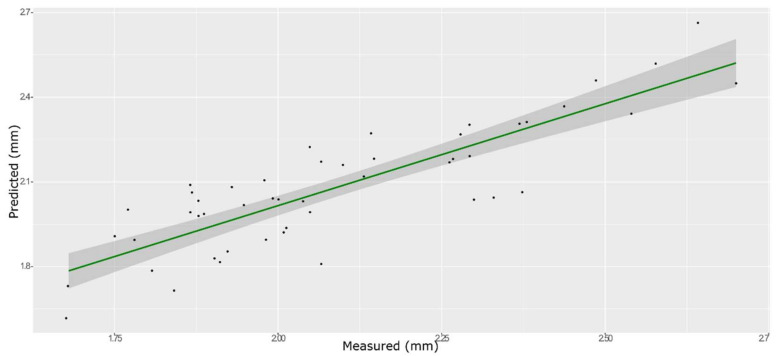
ANN model for BS on the test set, using the selected wavenumbers.

**Figure 9:**
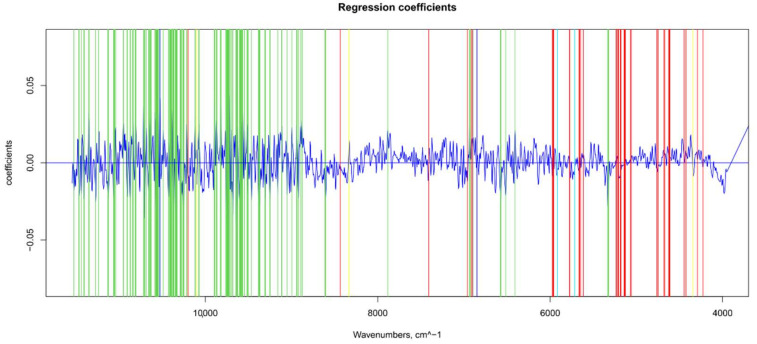
Βeta-coefficient values after outlier removal and mean center: values selected for ANN (in green), main NIR peaks for water (in blue), sugar and water (in light blue), sugar and organic acids (in yellow), and pectins (in red).

A total of 165 wavenumbers from high β-coefficients selection with a *p*-value < 0.05 were used as input data for the models. The performance of the ANN model strongly improved after the removal of the relatively uninformative variables, providing a better prediction over the PLS models (Figure 6, Figure 7 and Figure 8, and Supplementary Figures S4–S6 and Table 2). The β selection of optimal wavebands for the prediction of BS in grape berries resulted in better performing models compared to the MC-UVE. The selected wavenumbers include just two of the spectral areas, in which the overtones of sugars and water are usually found, produced by vibrational modes of O–H groups (Figure 9). Since the other signals produced by sugars are not among the selected wavelengths, we hypothesize that the influence of chemical composition on the BS is mainly attributable to compounds other than sugars. Therefore, it would be interesting to investigate the correlation of BS with the main molecules bearing hydroxyl functional groups found in grapes such as polyphenols, alcohols, and amino acids.

### 3.5. Hardness

**Figure 10:**
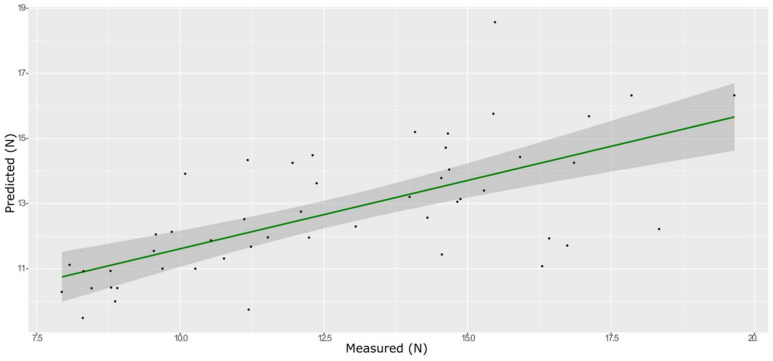
PLS model for BH on the test set, using the full spectral range.

**Figure 11:**
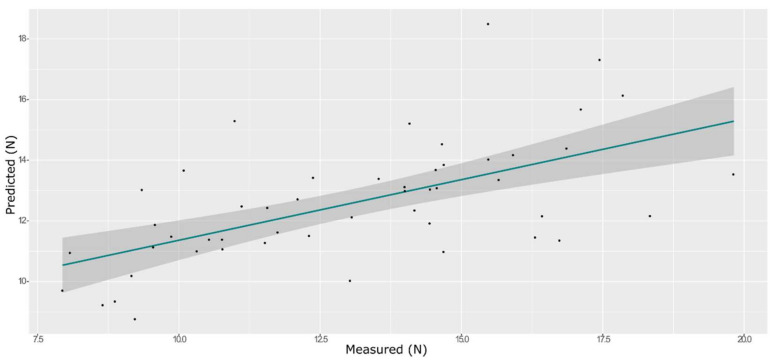
PLS model for BH on the test set, using the selected wavenumbers.

**Figure 12:**
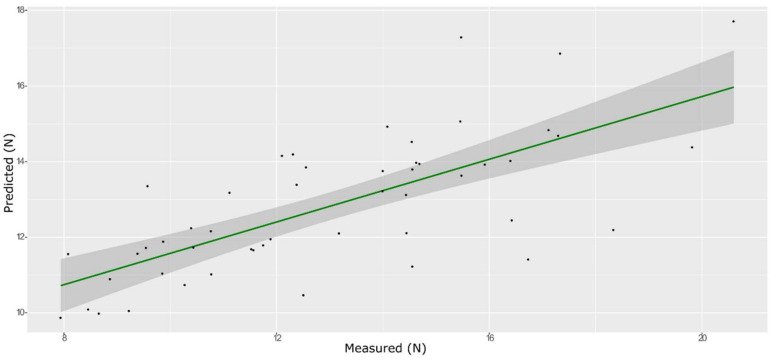
ANN model for BH on the test set, using the selected wavenumbers.

The model that obtained with mean-centered spectra provided the best prediction for the hardness parameter using both PLS (Figure 10 and Figure 11, and Supplementary Figures S7 and S8) and ANN (Figure 12 and Supplementary Figure S9). The best predictive performances were obtained from an ANN model built on 759 wavenumbers selected from high *β*-coefficients and with mean center as the pre-treatment (Table 3). The number of selected variables is more than three times higher than the input data used for the prediction of the other parameters. It is known that if the number of retained variables is too large, uninformative variables may be contained in the model and make its performance poor. However, the choice of the number of retained variables on this dataset was crucial in order to avoid over-fitting of the training set model, which was inevitable with each of the smaller sets of input data tested. However, even the best ANN model built with the TPA values for hardness on 759 selected wavelengths only showed screening abilities.

The ANN model provided the best prediction with a better fit, however it is worse compared to our previous models for the same parameter [11]. This is due to the lack of berry sorting in the present work. The performance of the developed models has been strongly influenced by the experimental variability. Sources of variability in the experimental design of a study can be divided into two categories, biological variability (due to the biological sample’s nature) and technical variability (due to measurement, instrumentation, and sample preparation) [44]. It is known that the NIR predictive ability in terms of accuracy and precision is strictly linked to the accuracy and precision of the primary reference method used [45]. In previous works, the importance of densimetric sorting of the berries before TPA testing was shown, since density greatly affects the texture properties of berries. The density class influences the berry hardness [43]; therefore, without any densimetric sorting, higher variability was expected. This is reflected in the prediction model’s accuracy and precision.

The ANN model built on not-sorted berries, however, fulfills a useful purpose since it can be used to assess the perceived grape crunchiness, not for quantitative purposes but for a qualitative fast screening.

**Figure 13:**
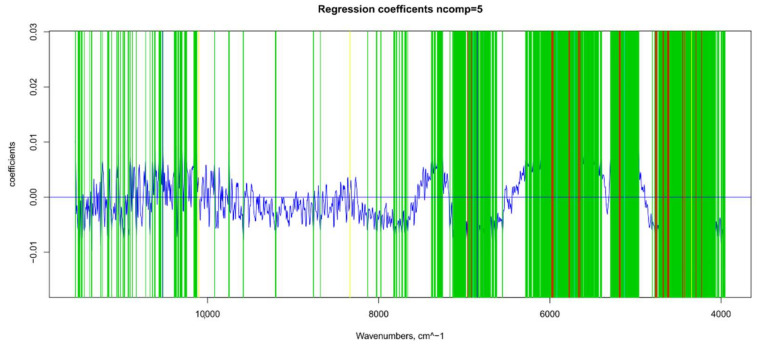
Βeta-coefficient values after outlier removal and mean center: values selected for ANN (in green), main NIR peaks for water (in blue), sugar and water (in light blue), sugar and organic acids (in yellow), and pectins (in red).

Interestingly, the peaks of pectins and water were all included in the wavelengths selected to build the model (Figure 13), while two main peaks linked to sugars and organic acids were discarded. This observation supports and corroborates the hypothesis of water and pectin contents’ influence on grape crunchiness [46,47].

### 3.6. Chewiness

Gumminess and chewiness are two alternative textural parameters. Gumminess is only applicable to semi-solids and is mutually exclusive with chewiness since a product would not be both a semi-solid and a solid at the same time [48]. Therefore, even though the texture analyzer produces a numeric outcome for gumminess and chewiness, we have only used the chewiness values for our grape samples.

The chewiness value (BCh as BH × BCo × BS) is a value (Joules) which is equal to force (Newtons) × distance (meters). Several authors have suggested that the influence of berry size on the force developed is of great importance. Indeed, the instrumental outcome of the TPA is often normalized using the berry diameter or the berry volume [43]. Therefore, we added the equatorial diameter values as an additional factor. 

**Figure 14:**
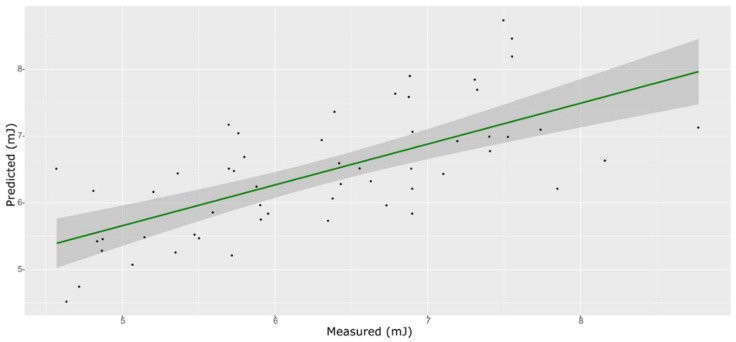
PLS model for BCh on the test set, using the full spectral range.

**Figure 15:**
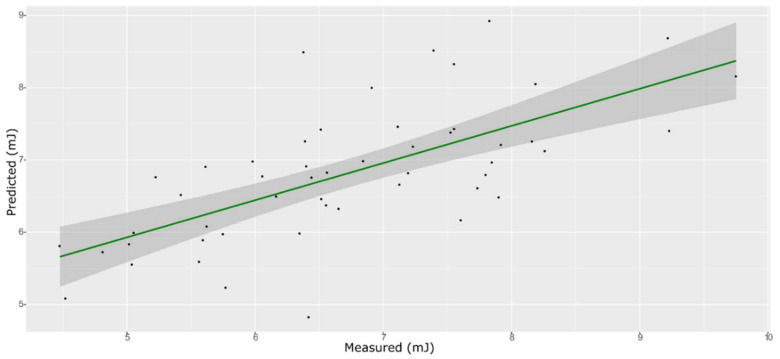
PLS model for BCh on the test set, using the selected wavenumbers.

**Figure 16:**
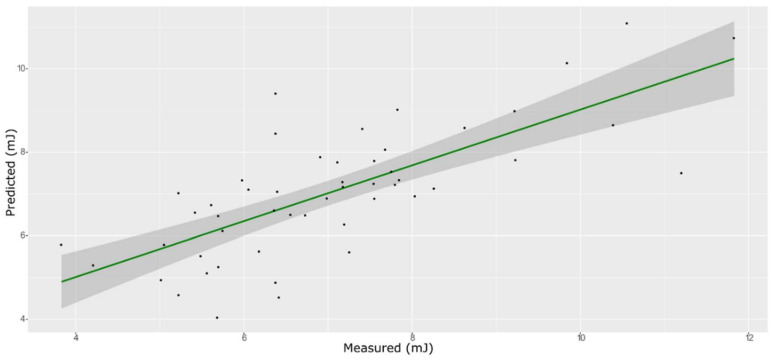
ANN model for BCh on the test set, using the selected wavenumbers.

**Figure 17:**
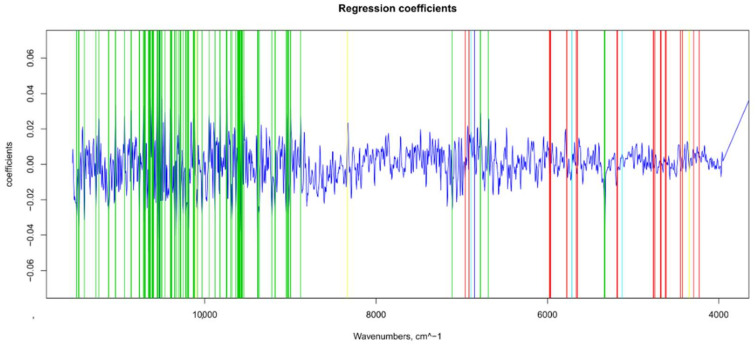
Βeta-coefficient values after outlier removal and mean center: values selected for ANN (in green), main NIR peaks for water (in blue), sugar and water (in light blue), sugar and organic acids (in yellow), and pectins (in red).

From the SNV pre-treated spectra, 116 wavenumbers were selected and used to build the best prediction model with an ANN. This model was superior to the best PLS models obtained with a mean center using all the wavenumbers or removing the uninformative ones (Figure 14, Figure 15 and Figure 16 and Supplementary Figures S10–S12, and Table 4). The water peak at 10,526 cm^−1^ is among the selected wavenumbers (Figure 17). The inclusion of water confirms previous findings showing that chewiness is a function of the moisture content in apple slices [49] and plays a critical role in the chewiness of bread. Indeed, both water and chewiness are lost with bread staling [50].

### 3.7. Cohesiveness 

Cohesiveness describes how well a food retains its form between the first and second chew. The BCo value is directly related to the compression strength of the internal bonds comprising the body of the food (due to the intermolecular attraction) [51]. Even the best models obtained for BCo had an R^2^ below 0.5 for both PLS and ANN models (data not shown). We hypothesize that the NIR spectra were not able to predict this parameter since the difference among our samples was too small. Indeed, the cohesiveness values show a small variability for values obtained with the texture analyzer (0.24 ± 0.03) and for BCo values divided by the equatorial diameter (0.012 ± 0.002).

## 4. Conclusions

In this article, NIR spectroscopy was applied to intact berries to predict textural parameters of table grape for fast screening. Together with the texture parameters, a chemical-related parameter (TSS) was measured for comparison. Besides the difficulties linked to the non-homogenous nature of the grape berries, an additional hurdle was the inevitable experimental variability arising from the lack of knowledge of the berry density class. The density class selection is compulsory before any instrumental texture measure, however, this is a time-consuming step we wanted to avoid. Unfortunately, an accurate quantification for all the textural parameters was not achieved without a density selection.

We obtained accurate quantitative models for springiness and sugar content and only qualitative models for hardness and chewiness. No satisfactory calibration model could be established between the NIR spectra and cohesiveness. However, previous articles found that the perceived cohesiveness is not accurately predicted by instrumental measures based on food rheological properties [51]. Therefore, we plan to build a prediction model for this parameter based on sensory data. 

Based on these results, it was concluded that NIR spectroscopy combined with an appropriate wavelength selection could be applied for a rapid preliminary screening of the main textural parameters of grape berries prior to other analyses.

## Figures and Tables

**Table 1:** Best performing models’ parameters for TSS.

Model	Spectra	R^2^	RMSE	Bias	RPD
PLS Entire spectrum (nComp 7)	Training with CV	0.69	1.02	0.004	2.33
	External validation	0.69	0.74	0.225	1.91
PLS Selected wavenumbers (nComp 4)	Training with CV	0.74	0.95	0.007	1.95
	External validation	0.46	0.87	−0.303	1.46
ANN	Training with CV	0.93	0.50	−0.132	3.66
	External validation	0.82	0.52	−0.048	2.35

nComp: number of selected components; CV: cross-validation; ANN: artificial neural networks.

**Table 2:** Best performing models’ parameters for BS.

Model	Spectra	R^2^	RMSE	Bias	RPD
PLS Entire spectrum (nComp8)	Training with CV	0.430	0.191	0.0010	1.33
	External validation	0.394	0.160	−0.0324	1.33
PLS Selected wavenumbers (nComp 2)	Training with CV	0.591	0.161	0.0005	1.57
	External validation	0.473	0.159	−0.0087	1.39
ANN	Training with CV	0.899	0.191	−0.1079	2.56
	External validation	0.724	0.133	−0.0094	1.94

nComp: number of selected components; CV: cross-validation.

**Table 3:** Best performing models’ parameters for BH.

Model	Spectra	R^2^	RMSE	Bias	RPD
PLS Entire spectrum (nComp 6)	Training with CV	0.44	2.49	−0.007	1.34
	External validation	0.44	2.32	−0.061	1.35
PLS Selected wavenumbers (nComp 8)	Training with CV	0.54	2.27	−0.002	1.49
	External validation	0.42	2.28	0.618	1.37
ANN	Training with CV	0.49	2.38	−0.022	1.40
	External validation	0.50	2.24	−0.198	1.41

nComp: number of selected components; CV: cross-validation.

**Table 4:** Best performing models’ parameters for BCh.

Model	Spectra	R^2^	RMSE	Bias	RPD
PLS Entire spectrum (nComp7)	Training with CV	0.545	1.096	0.0027	1.49
	External validation	0.383	0.790	−0.1506	1.31
PLS Selected wavenumbers (nComp 5)	Training with CV	0.604	1.002	−0.0053	1.59
	External validation	0.436	0.910	−0.0926	1.35
ANN	Training with CV	0.900	0.505	−0.2082	2.90
	External validation	0.530	1.176	0.0017	1.48

nComp: number of selected components; CV: cross-validation.

## Data Availability

Not applicable.

## Supplementary Materials

The following supporting information can be downloaded at: https://www.mdpi.com/article/10.3390/foods11030281/s1, Figure S1. PLS model for TSS on the training set using the full spectral range; Figure S2. PLS model for TSS on the training set using the selected wave numbers; Figure S3. ANN model for TSS on the training set; Figure S4. PLS model for BS on the training set using the full spectral range; Figure S5. PLS model for BS on the training set using the selected wave numbers; Figure S6. ANN model for BS on the training set using the selected wave numbers; Figure S7. PLS model for BH on the training set using the full spectral range; Figure S8. PLS model for BH on the training set using the selected wave numbers; Figure S9. ANN model for BH on the training set using the selected wave numbers; Figure S10. PLS model for BCh on the training set using the full spectral range; Figure S11. PLS model for BCh on the training set using the selected wave numbers; Figure S12. ANN model for BCh on the training set using the selected wave numbers.
