# Supplementary material for: Use of Artificial Neural Networks and NIR Spectroscopy for Non-Destructive Grape Texture Prediction
Source: Foods. 2022 Jan 20;11(3):281. doi: 10.3390/foods11030281 (PMC8834220; doi:10.3390/foods11030281)
Supplement: Supplementary file 1 [file foods-11-00281-s001.zip › foods-1531903-supplementary.pdf]

Supplementary material

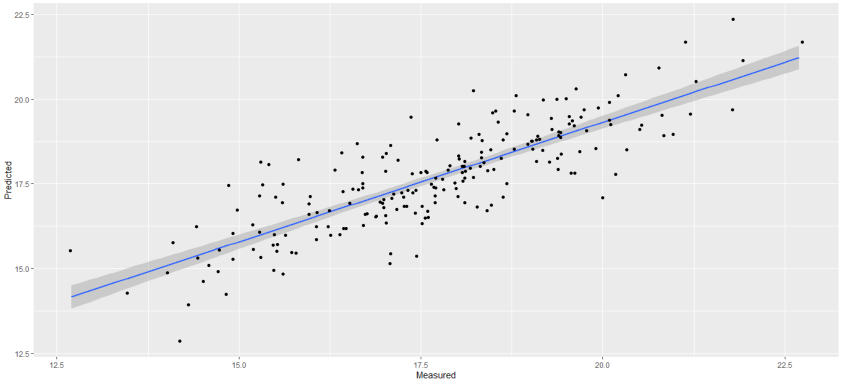

Figure S1. PLS model for TSS on the training stusing the full spectral range

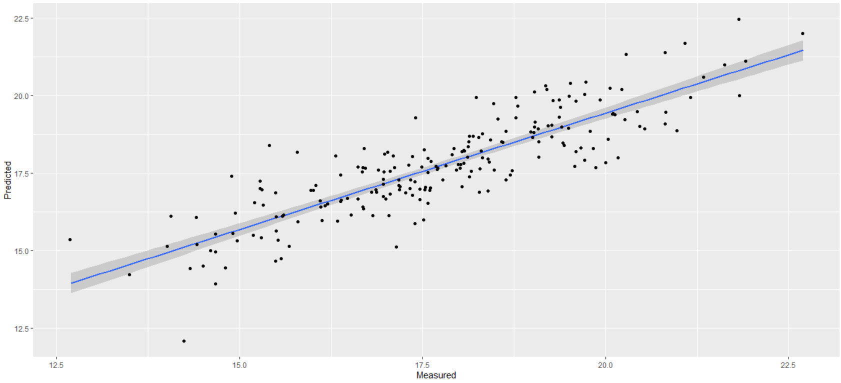

Figure S2. PLS model for TSS on the training set using the selected wave numbers

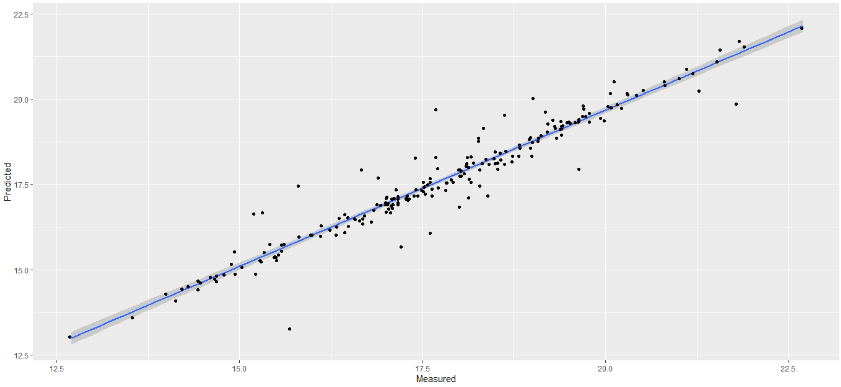

Figure S3. ANN model for TSS on the training set

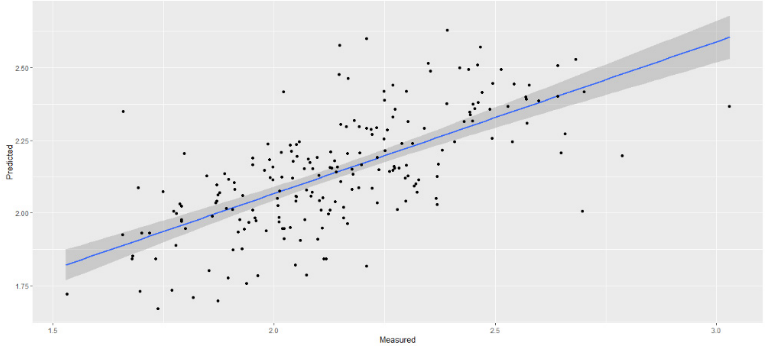

Figure S4. PLS model for BS on the training set using the full spectral range

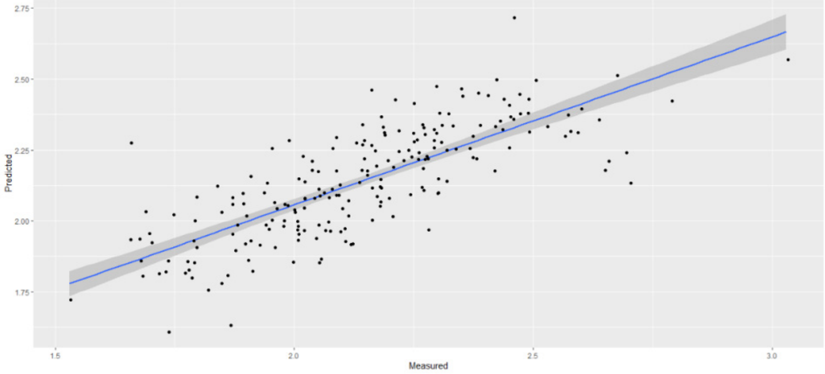

Figure S5. PLS model for BS on the training set using the selected wave numbers

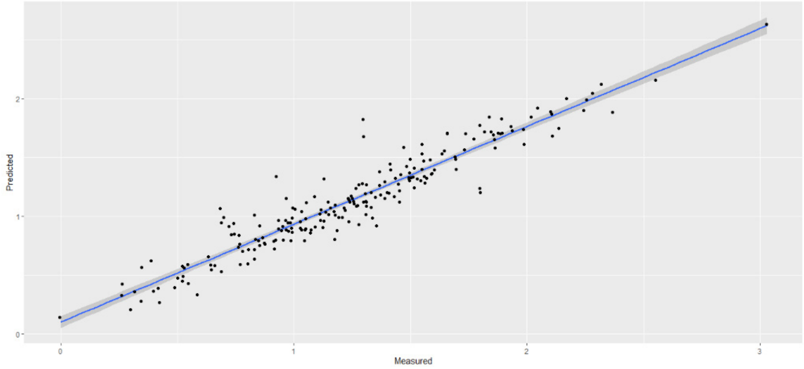

Figure S6. ANN model for BS on the training set using the selected wave numbers

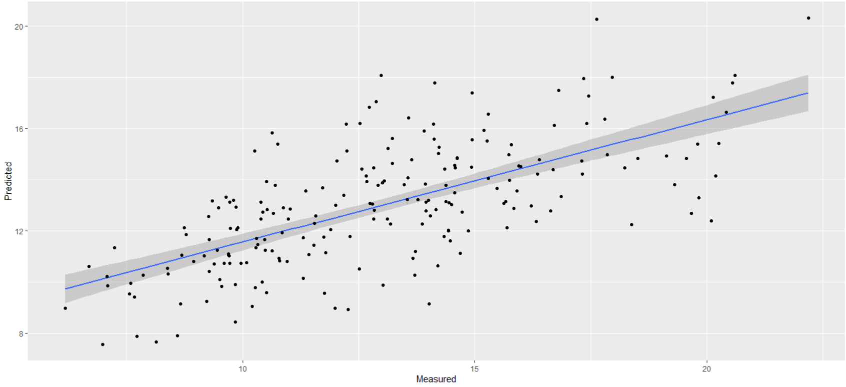

Figure S7. PLS model for BH on the training set using the full spectral range

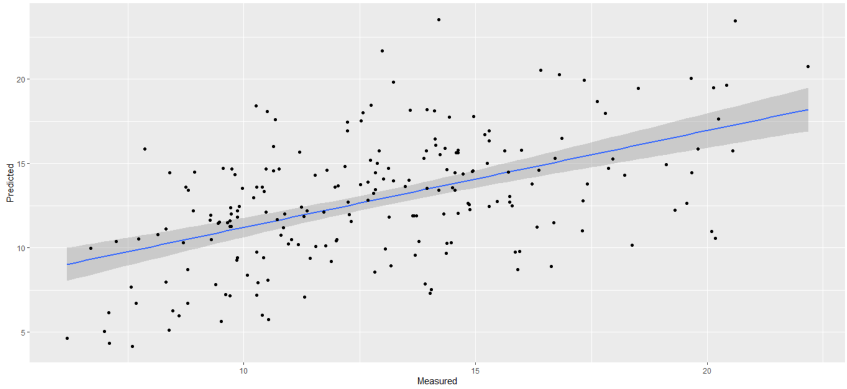

Figure S8. PLS model for BH on the training set using the selected wave numbers

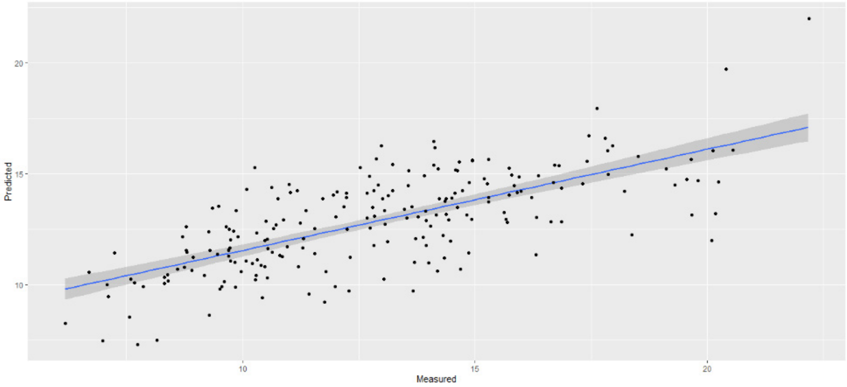

Figure S9. ANN model for BH on the training set using the selected wave numbers

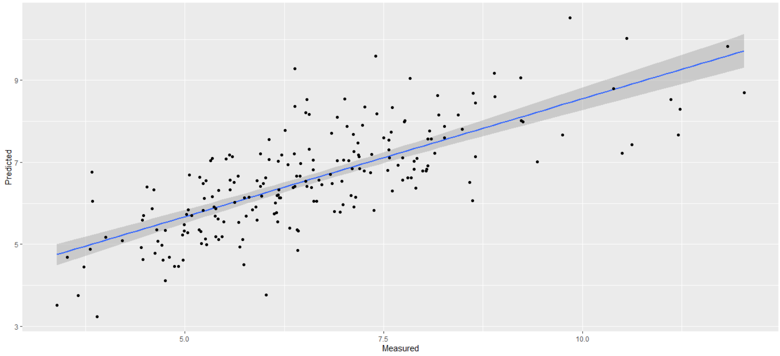

Figure S10. PLS model for BCh on the training set using the full spectral range

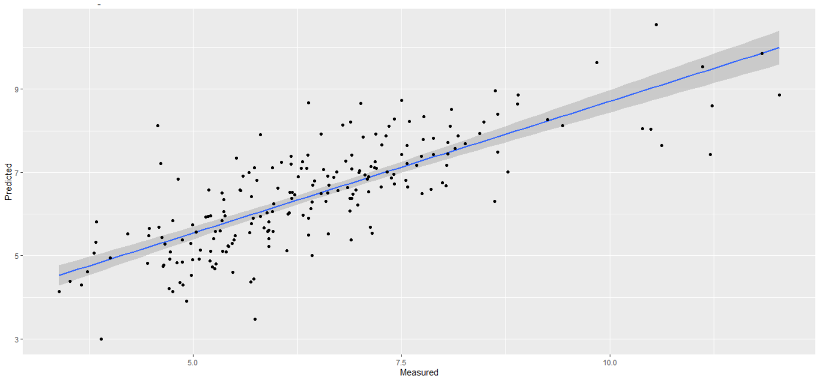

Figure S11. PLS model for BCh on the training set using the selected wave numbers

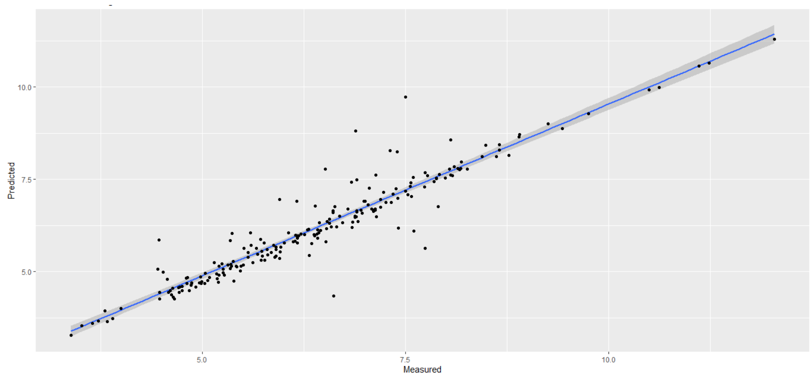

Figure S12. ANN model for BCh on the training set using the selected wave numbers
